# Scientific writing: a randomized controlled trial comparing standard and on-line instruction

**DOI:** 10.1186/1472-6920-9-27

**Published:** 2009-05-27

**Authors:** Amruta Phadtare, Anu Bahmani, Anand Shah, Ricardo Pietrobon

**Affiliations:** 1 Kalpavriksha Healthcare and Research, 301/A-wing, Saikrupa, Next to Ganesh Chhhaya, Anandnagar, Din Dayal Road, Dombivli West, Thane, 421202, Maharashtra, India; 2 University of Pennsylvania, Philadelphia and Duke University Medical Center, DUMC Box 3094, Durham, NC, 27710, USA; 3 Research on Research, Duke University Health System and Duke NUS Graduate Medical School in Singapore, DUMC Box 3094, Durham, NC 27710, USA

## Abstract

**Background:**

Writing plays a central role in the communication of scientific ideas and is therefore a key aspect in researcher education, ultimately determining the success and long-term sustainability of their careers. Despite the growing popularity of e-learning, we are not aware of any existing study comparing on-line vs. traditional classroom-based methods for teaching scientific writing.

**Methods:**

Forty eight participants from a medical, nursing and physiotherapy background from US and Brazil were randomly assigned to two groups (n = 24 per group): An on-line writing workshop group (on-line group), in which participants used virtual communication, google docs and standard writing templates, and a standard writing guidance training (standard group) where participants received standard instruction without the aid of virtual communication and writing templates. Two outcomes, manuscript quality was assessed using the scores obtained in Six subgroup analysis scale as the primary outcome measure, and satisfaction scores with Likert scale were evaluated. To control for observer variability, inter-observer reliability was assessed using Fleiss's kappa. A post-hoc analysis comparing rates of communication between mentors and participants was performed. Nonparametric tests were used to assess intervention efficacy.

**Results:**

Excellent inter-observer reliability among three reviewers was found, with an Intraclass Correlation Coefficient (ICC) agreement = 0.931882 and ICC consistency = 0.932485. On-line group had better overall manuscript quality (p = 0.0017, SSQSavg score 75.3 ± 14.21, ranging from 37 to 94) compared to the standard group (47.27 ± 14.64, ranging from 20 to 72). Participant satisfaction was higher in the on-line group (4.3 ± 0.73) compared to the standard group (3.09 ± 1.11) (p = 0.001). The standard group also had fewer communication events compared to the on-line group (0.91 ± 0.81 vs. 2.05 ± 1.23; p = 0.0219).

**Conclusion:**

Our protocol for on-line scientific writing instruction is better than standard face-to-face instruction in terms of writing quality and student satisfaction. Future studies should evaluate the protocol efficacy in larger longitudinal cohorts involving participants from different languages.

## Background

Scientific writing is the primary way used by researchers to communicate their findings and project ideas to peers and the general public. Essential for determining their career progress and sustainability, researchers might fail in situations where their science is good but not communicated in a clear and persuasive manner. [1-4] Although multiple previous studies have compared on-line and traditional training methodologies, [5-10] we are not aware of previous studies comparing different methodologies to the training of researchers in scientific writing.

Traditional methods of instruction such as classroom training, seminars and workshops, are inherently inflexible in terms of scheduling, location and customizability. Moreover, they are often perceived as outdated, boring, impersonal, and inapplicable to the real-world demands of the workplace. [11-15] Writing in collaboration tends to be superior to writing in isolation, as individual strengths are pooled and deficiencies improved, [16] having also been shown to increase publication rates among faculty members.[17] Although shared collegial authority is a feature of collaborative writing, [18] geographically dispersed teams face significant barriers related to dissent on content, hostility within the group and disagreement on strategy.[5] In addition, divergent backgrounds, asynchronous feedback, and communication issues all can affect a group's progress.[6]

The need for new instructional tools and techniques to overcome these limitations [19] has resulted in the rise of distance learning, e-learning, and virtual simulation. Distance education is promising since it is independent of time and place, reducing dissemination costs, multiplying learning opportunities, and eliminating travel time and related expenses.[19] For example, rather than having to follow a rigid class schedule, distance learners can receive course materials at home, enabling them to review materials and complete assignments with greater flexibility. Distance education is most useful when students and teachers are physically separated, requiring a technology-dependent interface.[19] As an extension of distance education, e-learning involves the delivery of course materials and the completion of assignments via the internet, frequently using streaming media (audio/video), live Webcasts, hyperlinked data and various other communication tools such as chat rooms, instant messaging, and videoconferencing.[20] A recent comparison of Web-based and face-to-face graduate curricula favored the Web-based course in terms of lower dropout rates and increased flexibility, affordability and attractiveness to students.[9] In addition, this evolving methodology allows for time/space flexibility, [21] wide accessibility, frequent contact among students and teachers, [7] and individual customization. [22] Potentially improving interaction, collaboration and feedback. Despite its advantages, Web-based learning has drawbacks, including feelings of isolation, frustration with unfamiliar practices, [8,20] lack of necessary infrastructure, and new costs associated with on-line courses.

Despite the paucity of evidence supporting its effectiveness in scientific writing instruction, a substantial increase in e-learning seems inevitable. It is therefore imperative to compare its efficacy with traditional instruction. This may ultimately contribute to the improvement of e-learning methods. Previous studies have compared Web-based and traditional classroom instruction. However, these studies have focused exclusively on student performance, [22,23] internet-based degree and certificate programs, [24] reactions of college staff to surveys, [25] and student grades.[26] They have also been applied narrowly to graduate-level courses in learning disabilities.[10] These studies did not address the specific issues surrounding on-line scientific writing instruction.

Our study used a randomized controlled trial design to compare on-line and traditional methods of instruction for training novice researchers in scientific writing. Outcomes included measurements of text quality, participant-mentor communication events and participant satisfaction.

## Methods

### Participants

Participants were recruited from second and third year programs in medical, nursing and physiotherapy schools in the United States and Brazil, all receiving informed consent prior to the initiation of study activities. The study was approved by the Institutional Review Board at Duke University and conducted between 2005 and 2007.

### Inclusion criteria

We recruited novice researchers with minimal previous scientific writing experience and no previous publications in Medline indexed journals. All eligible participants were enrolled, with no further exclusion criteria.

### Sample Size

Based on a pilot study of nine medical students writing manuscripts with our research group , we estimated a standard deviation of 15 points in the SSQS. Assuming a 20% difference between groups, the minimum sample size was estimated to be 22 participants per group, or 44 in all. To allow for attrition, our final sample size was set at 48.

### Randomization, Sequence Generation and Concealment

Random numbers were generated with GNU-R , using a 1:1 proportion, blocks of eight individuals, and stratification based on program of origin (nursing, physical therapy, and medicine). An initial randomization schedule was generated for 48 participants, followed by a sequence of size 15 to account for dropouts. Group assignments were placed in sealed envelopes and revealed after participants had signed informed consent. To ensure bliding, assignments were disclosed to analysts only after the results had been delivered. After stratified randomization, half of the pairs were assigned to the standard instruction course, while the other half were assigned to the on-line group.

### Interventions & Implementation

Participants were randomly assigned to one of two settings: standard writing guidance and an on-line writing workshop.

### On-line writing workshop group

**Figure 1:**
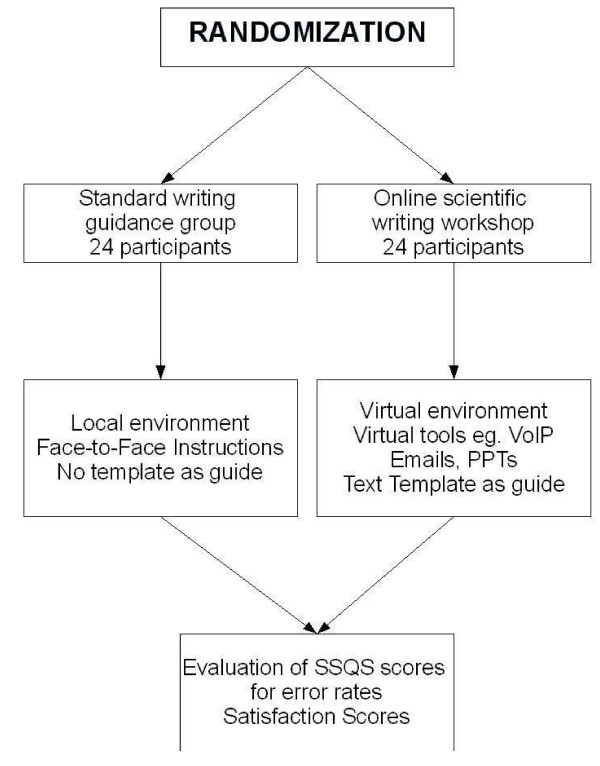
**Diagrammatic representation of the method implementation**.

In this group, the primary tools of instruction were PowerPoint presentations and audio conferences, supplemented by email, Google Docs and writing templates. Instructions for installing software, using Voice Over the Internet Protocol (VoIP) applications  and working with manuscript templates  were provided via email and PowerPoint presentations. Participants wrote their manuscripts using the Web-based word processor Writely (now Google Docs – ), and used VoIP applications, instant messaging and email to communicate with their mentors. After an initial session of instruction, mentors contacted students twice a week by email, with virtual meetings scheduled as needed. Students used customized writing templates, populating their manuscripts section by section in pre structured layouts. Templates were available on-line for reference. Google Docs, an open-access, Web-based word processor that enables collaboration among any number of researchers, anywhere in the world, was used to write, review, edit and share manuscripts. This application allows for synchronous as well as ashynchronous collaborative writing, allowing multiple users to edit the same source document, with a user-friendly interface. It archives previous versions along with details of the revision history, which allows users to compare versions of a document in different stages of editing. The on-line writing workshop group procedure is diagrammed in Figure 1.

### Standard writing guidance group

In this group, topics were assigned in a classroom setting. Participants could ask questions at the time of assignment, and they could communicate with instructors by email or conference call when necessary. Mentors, accessible by appointment, were assigned to each pair of participants. Together with their mentors they selected topics of mutual interest, sometimes complying with existing course requirements. Participants worked independently, using concepts and materials presented in class and consulting mentors by telephone. They used standard, computer-based word processing software such as Microsoft Word  or Open Office  to write their papers.

Mentor allocation, research question assignment and manuscript writing were executed in the same manner in both groups. In both groups, pairs of participants worked together to complete either the Introduction or Discussion section of a manuscript. On-line course materials were in English, while mentor support was provided in English and Portuguese.

### Outcomes and assessment strategies

Outcome variables consisted of manuscript quality and self-reported participant satisfaction. The quality of each manuscript was evaluated according to well-defined parameters using the Six-Subgroup Quality Scale (SSQS). [27] [see Additional file 1]. These parameters assessed the manuscripts' structure, application of research principles, and sequential flow of information. For manuscripts written in Portuguese, a bilingual researcher (RP) ensured that evaluations were cross-culturally consistent. Self-reported satisfaction with the overall training experience was measured using the Likert scale, with participants responding to statements on a scale of strongly disagreeing to strongly agreeing. Post hoc analysis evaluated the number of communication events (emails or phone calls) between participants and mentors. Evaluations were performed by three different reviewers. To ensure unbiased findings, statistical analysis was blinded; with analysts being unaware of which group participants were assigned to until the study analysis was complete.

### Quality of scientific writing

Manuscripts were analyzed by three expert reviewers: a nurse, a research technician, and a clinical epidemiologist. Prior to the study, reviewers participated in a preliminary session in which they rated ten examples of Introduction and Discussion sections using the SSQS scale. The scale included criteria such as focus; logical connection among portions of text; choice and arrangement of words (readable vs. awkward); writing mechanics (usage, grammar, spelling); content (engaged vs. uninvolved, acknowledgment of alternative points of view vs. single-mindedness); clarity of purpose and appropriateness of tone for the intended audience (clear vs. unclear purpose, language and tone appropriate and consistent); organization and development (support and elaboration, completeness, correct use of paragraphs); and style (sentence structure, concision, daring vs. safe). Disagreements were resolved by consensus. All criteria were graded on a scale of 1 to 5.

### Satisfaction scores

Self-reported satisfaction was measured using a 5-point Likert scale, participants responding to statements concerning their satisfaction with the course on a scale from "strongly disagree" to "strongly agree" (1 = Strongly disagree, 2 = Disagree, 3 = Neither agree nor disagree, 4 = Agree, 5 = Strongly Agree).

### Communication events

Communication events were defined as any interactions between participants and mentors, by email or phone, for the purpose of discussing manuscripts and receiving feedback. This outcome was considered secondary.

### Qualitative study

Using a convenience sampling method, we enrolled a subset of 16 novice researchers in a parallel qualitative study designed to identify the challenges encountered by participants in writing scientific manuscripts. They reported their progress using a prescribed methodology, described the problems they encountered, and identified their areas of greatest and least competence. Focus group discussions were transcribed using conventional qualitative analysis techniques. The results of this study have been reported elsewhere. [28]

### Covariates

Potential confounding factors included age, gender, marital status, course of study, previous experience with scientific writing, and choice of writing section (Introduction or Discussion).

### Statistical Methods

Calculations were performed using means and standard deviations for continuous variables, and frequencies and percentages for categorical variables. Efficacy was measured by analyzing the variance of SSSQ and satisfaction scores. SSQS scores were normalized from 0 to 100. An intention-to-treat protocol was preserved. We did not prevent students from employing other methods to improve their writing. Inter-observer reliability was assessed using Fleiss's kappa, then the two groups were compared by way of non-parametric tests for each domain of the SSQS scales using Intercooled Stata. Analysis was performed with GNU-R. A combination of kappa statistics was used to measure inter-observer reliability. This was followed by non-parametric tests comparing the groups' scores.

## Results

### Participant Flow

63 participants were initially enrolled after signing informed consent, of which 15 dropped out before randomization due to time constraints. The remaining 48 were randomly assigned to two groups of 24 each.

### Recruitment

Participants were recruited between September 2005 and July 2006, with follow-up ending in November 2007.

### Baseline data

There were no statistically significant differences between groups with respect to age, gender, marital status, course of study, choice of writing section or previous experience (Table 1). Participants were balanced regarding gender distribution, mostly single, and with no previous experience writing scientific manuscripts. Most participants used the Introduction as the section used for participation in this study.

**Table 1:** Baseline characteristics

	**Mean(Standard Deviation)** **[Range Values]**		
**Baseline characteristics**	**on-line scientific writing workshop**	**Standard writing guidance**	**P value**
**Age**	23.54(+/- 1.50) [9,10,23,27,29,30]	23.17(+/- 1.55) [9,10,23,27,29,30]	0.9248
**Gender**			0.6892
Female	13/48(27.08%)	14/48(29.17%)	
Male	11/48(22.92%)	10/48(20.83%)	
**Marital status**			0.4701
Married	1/48(2.08%)	1/48(2.08%)	
Single	23/48(47.92%)	23/48(47.92%)	
**Course**			0.768
Med	13/48(27.83%)	14/48(29.16%)	
Nursing	6/48(12.5%)	5/48(10.42%)	
PT	5/48(10.42%)	4/48(8.33%)	
**Previous experience research writing**			0.574
No	16/48(33.33%)	18/48(37.5%)	
Yes	8/48(16.66%)	5/48(10.42%)	
**Manuscript section**			
Introduction	16/48 (33%)	13/48 (27%)	0.685
Discussion	11/48 (22.0%)	8/48 (16.6%)	0.544

### Outcomes and Estimations

Excellent inter-observer reliability was found among the three reviewers, with an Intraclass Correlation Coefficient of 0.93. Writing quality measured by the average SSQS for the on-line group (75.3 ± 14.21, ranging from 37 to 94) was lower than that of the standard group (47.27 ± 14.64, ranging from 20–72) (p = 0.0017), and participants in the on-line group were more satisfied with the experience (4.3 ± 0.73, ranging from 3 to 5) than their counterparts in the traditional group (3.09 ± 1.11, ranging from 1 to 5) (p = 0.001).

Post hoc analysis revealed that participants in the traditional group reported fewer communication events (0.91 ± 0.81) than the on-line group (2.05 ± 1.23; p = 0.0219) (Table 2). Also, participants with previous experience in scientific writing made fewer errors than participants with no experience (63.82 ± 18.60 vs. 59.48 ± 20.78 respectively; p = 0.002). Reported satisfaction and quantity of communication were not significantly affected by previous experience. For all other variables, including as gender, marital status, course of study, choice of writing section and attitude toward the writing process, the two groups were comparable and there were no significant differences (Table 3).

**Table 2:** Association between the two groups and the outcomes

	**Mean** **(Standard Deviation)** **[Range Values]**	**Mean** **(Standard Deviation)** **[Range Values]**	
		
**Outcome**	**on-line scientific writing workshop**	**Standard writing guidance**	**P value**
**SSQSavg**	75.3(+/- 14.21) [37–94]	47.27(+/- 14.64) [20–72]	0.0017
**Satisfaction with method**	4.3(+/- 0.73) [3-5]	3.09(+/- 1.11) [1-5]	< 0.001
**Number communication events with mentor**	2.05(+/- 1.23) [1-6]	0.91(+/- 0.81) [0–3]	0.0219

**Table 3:** Post hoc analysis of outcomes and other variables

		**Number communication events**		**Satisfaction**	
		
**Predictors**	P-values	Mean (Standard Deviation) Range Values	P-values	Mean (Standard Deviation) Range Values	P-values
**Enjoys writing**	0.5881		0.972		0.8264
1		1 +/-(1) 0–2		2.67+/-(1.52) 1–4	
2		1.14 +/-(0.69) 0–2		3.71+/-(1.49) 1–5	
3		1.94 +/-(1.43) 0–6		3.82+/-(0.95) 0–2	
4		1.08 +/-(0.99) 0–3		3.67+/-(0.98) 2–5	
5		1.33 +/-(0.57) 1–2		3.67+/-(1.52) 2–5	
**Gender**	0.1028		0.3819		0.8127
Female		1.52 +/-(1.32) 0–6		3.6 +/-(1.22) 1–5	
Male		1.35 +/-(0.93) 0–4		3.76+/-(0.97) 2–5	
**Marital status**	0.289		0.9655		0.7156
Married		1.5 +/-(0.70) 1–2		4 4–4	
Single		1.45 +/-(1.19) 0–6		3.65+/-(1.14) 1–5	
**Course**	0.5727		0.4723		0.05372
Med		1.44 +/-(1.04) 0–4		3.4 +/-(1.08) 1–5	
Nursing		1.87 +/-(1.88) 0–6		4.25+/-(0.70) 3–5	
PT		1.11 +/-(0.60) 0–2		3.89+/-(1.36) 1–5	
**Previous experience research writing**	0.002		0.1046		0.3920
No		1.29 +/-(1.00) 0–4		3.61+/-(1.05) 1–5	
Yes		1.91 +/-(1.51) 0–6		3.82+/-(1.32) 1–5	

## Discussion

Our study found that the on-line scientific writing group performed significantly better than the standard writing guidance group in terms of writing quality, also reporting greater overall satisfaction. The on-line group also reported a greater number of participant-mentor communication events in the post hoc analysis. Because this was not the primary objective of this study, it should be investigated further to understand the effects of on-line learning on collaboration and group instruction. A significantly lower error rate occurred among participants with previous scientific writing experience, but reported satisfaction was unaffected by writing experience. Inter-observer reliability for the results of this study was high. The use of Google Docs clearly enhanced participants' familiarity with an increasingly popular method of collaboration, as well as improving the mentors' efficiency.

Web-based education has become increasingly popular over the past decade. Multiple studies have evaluated the effectiveness of Web-based and other computer-assisted teaching methods. Although they have mainly addressed distance education, [23,27,29] broad comparisons to this study are possible, as distance education often includes Web-based or other computer-assisted education. Significant advantages of on-line teaching methods over traditional classroom methods have been previously demonstrated, [28,30-33] on-line instruction producing enhanced performance, [30,34] cognitive gains and higher satisfaction, [32] and improved test scores. [31] For example, in one study [31] students who were provided with Web-based educational materials obtained higher scores than those who were not provided with similar materials. It has also been shown that computer environments are conducive to the presentation of visual material that tends to benefit students. [29,35,36] Nurses have shown a greater willingness to adopt Web-based methodologies, [37] and they consider the benefits to far outweigh their disadvantages.48

Although studies favoring traditional teaching over on-line methods do exist, [23] studies yielding neutral results are more prevalent. A comparison of the two methods in graduate-level courses in learning disabilities and other curricula evaluated quality, test scores, cognitive gains, and student performance, success and satisfaction, finding no significant differences between distance education using on-line materials and traditional classroom-based instruction. [10,22,26,38] Since on-line learning is becoming increasingly prevalent due to its easy scalability and flexibility in scheduling, even despite its current drawbacks, on-line teaching methods will likely improve with the emergence of new Web-based technologies and thus need constant re-evaluation.

Student satisfaction is an important consideration when implementing new teaching methods and the present study finds greater satisfaction with on-line workshops. Carr, [39] however, found that student satisfaction was lower in Web-based distance education despite higher rates of success. The author attributed this result to technical difficulties, including problems with internet connection and computer problems requiring the assistance of engineers, which ultimately resulted in greater time expenditure by students in the distance learning environment. Finally, on-line courses can also automate processes such as test score collation, saving time by reducing faculty workload and thus providing students with faster feedback. [40]

Student-mentor communication was higher in the Web-based group, indicating a more open atmosphere for comments and criticism. Earlier studies evaluating students' reactions toward distance education have documented feelings of loneliness and frustration, and have cautioned against a shift away from traditional modes of group learning. [41] However, this trend is likely to change as students that have grown up surrounded by Web applications as part of their life enter distance learning programs. These students are more likely to offer critical suggestions, facilitating beneficial changes to the curriculum. [42,43]

Mixed results for performance advantages and decision making have been noted on a variety of tasks in computer mediated versus face-to-face learning environments. [44] Contributors to these mixed results included factors such as participants' prior experience with on-line courses, their grades, [34,37] computer competency, [45] and idiosyncratic interactions with a given system of instruction as well as instructor skills, [46] the presence or absence of supervision, [47] and the relatively slow diffusion of Web-based technology as described by Karl Pajo and Catherine Wallace. [25] Web-based learning is a maturing technology, and early studies may have suffered from poor program design and students' lack of familiarity with on-line environments, as shown in a study by Spooner. [29] This study analyzed student response to the two types of instruction, and, interestingly, results for one parameter (class organization) were contradictory: Traditional methods were rated superior in one of the two courses studied, while distance education was favored in the other.

The use of Blackboard™ , a Web-based learning system, has been proven effective in helping students write research papers. [48] However, in this system, access is limited to registered candidates or institutions, preventing collaboration with outside researchers who might be working on the same project. Also, while the system is useful for tracking students' use of course materials and monitoring their progress, it is not open-source and can be costly to implement. The present study used Google Docs , which is a freely available interface for sharing, editing, and tracking on-line documents. We are not aware of any existing previous study making use of Google Docs for the purposes of scientific writing. Scientific writing demands collaboration, in the form of back-and-forth communication with collaborators, peers, mentors and outside researchers. Given this requirement, Google Docs is an excellent solution that precludes the time-consuming compiling of multiple iterations of text that occurs with the exchange of documents via email or hard copies.

Despite significant advances in relation to the previous literature in the field, our study has limitations. First, the number of potential confounding factors we could track was limited, and did not include subjects' prior participation in on-line courses, their grades, [34,37] proficiency with computers, [45] and interaction with the system of instruction, as well as instructor skills [46] and the presence or absence of supervision. [47] Examining these factors was beyond the scope of this study. Second, our mentors were not blinded, which may have resulted in bias. Because it was not possible to maintain blinding in the study's early stages, we ensured that the final statistical analysis was blinded for an unbiased interpretation of the results. Third, our study did not evaluate participants' perceptions of their own performance. Several earlier studies have demonstrated that, although the two methods are comparable in terms of performance, [[9,36,42,43], and [49]] the same consistency is not seen in the participants' perceptions of their own performance. [50-52] While the present study did not include a parameter for self-assessment, reports of satisfaction provide a rough correspondence. Last, our current study used the SSQS scale for measuring manuscript quality without this scale having a formal cross-cultural validation. As we did not have a large enough sample to conduct a stratified analysis, it is unclear whether language might have affected our outcomes.

Future studies should address the problems associated with using imperfect measurement scales such as SSQS, which is prone to subjective bias, and tools should be developed to objectively measure writing quality. The lack of tools to objectively evaluate manuscript quality and participants' self-assessment makes it difficult to interpret the results of this type of study. Thus, developing tools that consistently evaluate participants' perceptions of their own performance, as well as objective measurements of the quality of scientific writing, could prove beneficial. Several other factors are also found to influence the outcome of this type of study, including participants' previous experience with on-line courses, their grades, [34,37] computer competency, [45] and interaction with the system in question, as well as instructor skill [46] and the presence or absence of supervision. [47] Future studies should enroll participants in sufficient numbers to stratify comparison groups according to these factors.

## Conclusion

This study suggests that the on-line scientific writing methodology was superior to traditional classroom-based instruction, which suggests that reservations concerning Web-based instruction should be reconsidered. Web-based workshops also resulted in reports of greater satisfaction among participants, although the generalization of this result should be tested in future studies. We therefore strongly encourage the use of on-line environment to provide a highly scalable method to educate the next generation of biomedical research.

## Competing interests

The authors declare that they have no competing interests.

## Authors' contributions

AP-participated in manuscript writing and critical analysis of results. AB-participated in manuscript writing and critical analysis. AS-performed data analysis, participated in study design and manuscript review. RP-designed and conducted the study and reviewed the manuscript. All authors read and approved the final manuscript.

## Pre-publication history

The pre-publication history for this paper can be accessed here:



## Supplementary Material

Additional file 1 **Six Sub Group Quality Scale**. This scale provided was used to assess manuscript quality, which was a primary outcome. Click here for file

## References

[B1] Peh WCG (2007). Scientific writing and publishing: its importance to radiologists. Biomed Imaging Interv J.

[B2] Rothman K (1998). Writing for epidemiology. Epidemiology.

[B3] David AS (1990). How to do it: write a classic paper. BMJ.

[B4] Day R, Gastel B (2006). How to write and publish a scientific paper.

[B5] Hinds PJ, Bailey DE (2003). Out of sight, out of sync: Understanding conflict in distributed teams. Organization Science.

[B6] Gennari JH, Weng C, Benedetti J, McDonald DW (2005). Asynchronous communication among clinical researchers: A study for systems design. Int J Med Inform.

[B7] Cole RA (2000). Issues in Web-based pedagogy: A critical primer.

[B8] Cook D (2007). Web-based learning: pros, cons and controversies. Clin Med.

[B9] Horiuchi S, Yukari Y, Miki K, Yumi S, Kazuhiro N (2008). Evaluation of a Web-based graduate continuing nursing education program in Japan: A randomized controlled trial. Nurse Education Today.

[B10] Tinnerman L (2006). A Comparative Study Between Traditional and Distance Education Instructional Environments Involving Two Graduate Level Learning Disabilities Classes. International Journal of Instructional Technology and Learning.

[B11] Diamond R (1997). Broad curriculum reform is needed if students are to master core skills. Chronicle of Higher Education.

[B12] Gardiner LF (1997). Producing dramatic increases in student learning: Can we do it?. National Teaching and Learning Forum.

[B13] Charles H (1998). A proper education. Change.

[B14] Roueche JE (1998). American Imperative-Essay, Virtual Library".

[B15] Wingspread Group on Higher Education (1993). An American Imperative: Higher Expectations for Higher Education.

[B16] Group work and collaborative writing. http://dhc.ucdavis.edu/vohs/sec11.html.

[B17] Grzybowski SCW, Bates J, Calam B, Alred J, Martin RE, Andrew R, Rieb L, Harris S, Wiebe C, Knell E, Berger S (2003). A physician peer support writing group. FamMed.

[B18] Galligan L, Cretchley P, George L, Martin McDonald K, McDonald J, Rankin J (2003). Evolution and emerging trends of university writing groups. Queensland Journal of Educational Research.

[B19] Glenn AS (2001). A Comparison of Distance Learning and Traditional Learning Environments. http://www.eric.ed.gov/ERICWebPortal/custom/portlets/recordDetails/detailmini.jsp?_nfpb=true&_&ERICExtSearch_SearchValue_0=ED457778&ERICExtSearch_SearchType_0=no&accno=ED457778.

[B20] McKimm J, Jollie C, Cantillon P (2003). ABC of learning and teaching: Web based learning. BMJ.

[B21] Takiya S, Archbold J, Berge ZL (2005). Flexible training's intrusion on work/life balance. Turkish on-line Journal of Distance Education.

[B22] Mehlenbacher B, Miller CR, Covington D, Larsen JS (2000). Active and interactive learning on-line: a comparison of Web-based and conventional writing classes. Professional Communication, IEEE Transactions.

[B23] Sawyer T (1998). The effects of computerized and conventional study guides on achievement in college students. Journal of Computer-Based Instruction.

[B24] Wallace DR, Mutooni P (1997). A comparative evaluation of World Wide Web-based and classroom teaching. J Eng Educ.

[B25] Pajo K, Wallace C (2001). Barriers to the uptake of Web-based technology by university teachers. Journal of Distance Education/Revue de l'enseignement à distance.

[B26] Roach V, Lemasters L (2006). Satisfaction with on-line learning: A comparative descriptive study. Journal of Interactive on-line Learning.

[B27] Ransdell S, Levy CM, Levy CM, Ransdell S (1996). Working memory constraints on writing quality and fluency. The science of writing.

[B28] Shah J, Shah A, Pietrobon R (2009). Scientific writing of novice researchers: what difficulties and encouragements do they encounter. Academic medicine: Journal of the Association of American Medical Colleges.

[B29] Spooner F, Jordan L, Algozzine B, Spooner M (1999). Student ratings of instruction in distance learning and on-campus classes. Journal of Educational Research.

[B30] Smeaton A, Keogh G (1999). An analysis of the use of virtual delivery of undergraduate lectures. Computers and Education.

[B31] Ochoa J (2008). Randomized Comparison Between Traditional and Traditional Plus Interactive Web-Based Methods for Teaching Seizure Disorders. Teach Learn Med.

[B32] Jeffries P (2001). Computer versus lecture: A comparison of two methods of teaching oral medication administration in a nursing skills laboratory. Journal of Nursing Education.

[B33] Griffin J (2003). Technology in the teaching of Neuroscience: enhanced students learning. ADV PHYSIOL EDUC.

[B34] Dutton J (2005). Characteristics and Performance of Students in an on-line Section of Business Statistics. Journal of Statistics Education.

[B35] Pietrobon R, Guller U, Martins H, Menezes AP, Higgins LD, Jacobs DO (2004). A suite of Web applications to streamline the interdisciplinary collaboration in secondary data analyses. BMC Medical Research Methodology.

[B36] Welsh J, Null C (1991). The effects of computer-based instruction on college students' comprehension of classic research. Behavior Research Methods, Instruments and Computers.

[B37] Huckstadt A (2005). Evaluation of interactive on-line courses for advanced practice nurses. J Am Acad Nurse Pract.

[B38] Schoech D, Helton D (2002). Qualitative and quantitative analysis of a course taught via classroom and internet chatroom. Qualitative Soc Work.

[B39] Sarah C (2000). on-line psychology instruction is effective, but not satisfying, study finds. The Chronicle of Higher Education.

[B40] Anderson H (2005). On-line Student Course Evaluations: Review of Literature and a Pilot Study. American Journal of Pharmaceutical Education.

[B41] Hara N, Kling R (1999). Student's frustrations with a Web-based distance education course. First Monday.

[B42] Layne BH, DeCristofor JR, McGinty D (1999). Electronic versus traditional student ratings of instruction. Res Higher Educ.

[B43] Kasiar JB, Schroeder SL, Holstad SG (2001). Comparison of traditional and Web-based course evaluation processes in a required, team-taught pharmacotherapy course. Am J Pharm Educ.

[B44] Luppicini R (2007). Review of computer mediated communication research for education. Instructional Science.

[B45] Wojciechowski A (2005). Individual Student Characteristics: Can Any Be Predictors Of Success In on-line Classes?. Journal of Distance Learning Administration.

[B46] Wellman G (2005). Comparing Learning Style to Performance in On-Line Teaching: Impact of Proctored vs. Un-Proctored Testing. Journal of Interactive on-line Learning.

[B47] Meyer K (2003). Face-to-Face versus threaded discussions:The role of time and higher order thinking. JALN.

[B48] Stone V (2004). Delivery of Web-based instruction using Blackboard: a collaborative project. J Med Libr Assoc.

[B49] Russell T (1999). The No Significant Difference Phenomenon.

[B50] Schoech D (2000). Teaching over the Internet: Results of one doctoral course. Research on Social Work Practice.

[B51] Sonner BS (1999). Success in the capstone business course – Assessing the effectiveness of distance learning. Journal of Education for Business.

[B52] Bower B (2001). Distance education: Facing the faculty challenge. Journal of Distance Learning Administration.

