# Supplementary material for: Scientific writing: a randomized controlled trial comparing standard and on-line instruction
Source: BMC Med Educ. 2009 May 27;9:27. doi: 10.1186/1472-6920-9-27 (PMC2701428; doi:10.1186/1472-6920-9-27)
Supplement: Additional file 1 — Six Sub Group Quality Scale. This scale provided was used to assess manuscript quality, which was a primary outcome. [file 1472-6920-9-27-S1.doc]

**Additional File 1**

**Title: Six Sub Group Quality Scale.
Description: This scale provided was used to assess manuscript quality, which was a primary outcome.**

QUALITY

| **Item**  **no** | **Statement** | **Scoring** |
| --- | --- | --- |
|  | **Subgroup 1 -- Words: Choice and Arrangement** |  |
|  | **1. Readable vs. Awkward** |  |
| 5 | All sentences are crystal clear on first reading--it is not necessary to pause and think |  |
| 4 | All sentences are clear on first reading, but it is sometimes necessary to pause and think |
| 2 | More than one sentence must be reread to derive meaning; writer intent is ambiguous |
| 1 | After repeated readings, writer intent is still unknown |
|  | **Subgroup 2 -- Technical Quality: Mechanics** |  |
|  | **2. Tenses** |  |
| 5 | All tenses correctly used |  |
| 4 | Most tenses correctly used |
| 2 | Most tenses incorrectly used |
| 1 | Consistent errors in tense |
|  | **3. Grammar** |  |
| 5 | Almost no grammatical errors |  |
| 4 | Few grammatical errors |
| 2 | Many grammatical errors |
| 1 | Little evidence of grammatical knowledge, very poor grammar |
|  | **4. Spelling** |  |
| 5 | No misspelled words |  |
| 4 | One misspelled word |
| 2 | Two or more misspelled words |
| 1 | Two or more misspelled words, including common words (elementary school level) |
|  | **Subgroup 3 -- Content of Essay** |  |
|  | **5. Engaged vs. Uninvolved** |  |
| 5 | Extremely serious or passionate and very engaged with the subject; formal, no redundancies, no use of inappropriate jokes |  |
| 4 | Not quite as engaged or passionate, while still formal and not redundant; serious, with almost no use of inappropriate jokes |
| 2 | Some inappropriate jokes and not very serious; casual and unengaged |
| 1 | Many inappropriate jokes and a clear lack of interest in the topic; very redundant, casual and uninvolved |
|  | **6. Alternative Points vs. Egocentric** |  |
| 5 | Consistent acknowledgment and discussion of other points of view |  |
| 4 | Some acknowledgment and discussion of other points of view |
| 2 | Little acknowledgment and almost no discussion of other points of view, vehement and close-minded |
| 1 | Almost no acknowledgment and lack of discussion of other points of view, very vehement and close-minded |
|  | **Subgroup 4 - Purpose/Audience/Tone** |  |
|  | **7. Purpose Clear vs. Unclear** |  |
| 5 | Definite statement of purpose and at least 75% of body revolves around the thesis |  |
| 4 | 50-75% of body revolves around the statement of purpose |
| 2 | No statement of purpose but some coherence |
| 1 | No statement of purpose, scattered points |
|  | **8. Language and Tone Appropriateness/Consistency** |  |
| 5 | Respectful, formal, without sarcasm; near-total consistency |  |
| 4 | Respectful, formal, almost no sarcasm or double meanings; almost no inconsistency |
| 2 | Some sarcasm or jokes; casual; half the essay uses different language and/or tone than the other half |
| 1 | Very casual, bordering on disrespectful; sarcastic and/or jokey; totally disconnected |
|  | **Subgroup 5 - Organization and Development** |  |
|  | **9. Support and Elaboration** |  |
| 5 | Many different reasons and at least 65% are elaborated |  |
| 4 | Few reasons and at least 65% are elaborated |
| 2 | Many reasons and less than 65% are elaborated |
| 1 | Few reasons and less than 65% are elaborated |
|  | **10. Sense of Completeness** |  |
| 5 | All thoughts or ideas tied together by one or more conclusions |  |
| 4 | Most thoughts or ideas tied into a conclusion |
| 2 | No general conclusions but thoughts have closure |
| 1 | No conclusions |
|  | **11. Paragraphing** |  |
| 5 | Sufficient use of paragraphs to separate ideas; paragraphs contain opening sentence and some elaboration, and end with a sentence that leads smoothly into the next paragraph |  |
| 4 | Sufficient use of paragraphs to separate ideas;  paragraphs contain opening sentence and some elaboration; choppy transitions between paragraphs |
| 2 | Insufficient use of paragraphs; only 1 or 2 paragraphs used when 5 or 6 are needed, or excessive number of of paragraphs; almost no opening sentences; choppy transitions |
| 1 | No paragraphs or paragraphs are arbitrary |
|  | **Subgroup 6  - Style** |  |
|  | **12. Sentence Structure and Conciseness** |  |
| 5 | Almost no run-on sentences or wasted words; clear and to the point |
| 4 | Almost no run-on sentences; few wasted words |
| 2 | Redundant and immature; not clear or concise |
| 1 | Many wasted words and run-on sentences; immature; very difficult to read |
|  | **13. Daring vs. Safe** |  |
| 5 | Unique ideas and very mature, creative, extensive use of language |  |
| 4 | Unique ideas or very mature, creative, extensive use of language |
| 2 | No new thoughts and moderate use of language |
| 1 | No new thoughts and/or simple language with limited vocabulary |
|  |  |  |
